# Sex differences, chronobiology and general anaesthesia in activities of the autonomic nervous system in rats

**DOI:** 10.1113/EP091143

**Published:** 2023-03-23

**Authors:** Pavol Svorc, Pavol Svorc, Sona Gresova

**Affiliations:** ^1^ Department of Physiology and Pathophysiology, Faculty of Medicine Ostrava University Ostrava Czech Republic; ^2^ Department of Physiology, Faculty of Medicine Safarik University Košice Slovak Republic

**Keywords:** chronobiology, HRV, rats, sex

## Abstract

Heart rate variability (HRV) is commonly used in experimental studies to assess sympathetic and parasympathetic activities. The belief that HRV in rodents reflects similar cardiovascular regulations in humans is supported by evidence, and HRV in rats appears to be at least analogous to that in humans, although the degree of influence of the parasympathetic division of the autonomic nervous system (ANS) may be greater in rats than in humans. Experimental studies are based on control or baseline values, on the basis of which the change in ANS activity after a given experimental intervention is assessed, but it is known that the ANS in rats is very sensitive to various stress interventions, such as the manipulation itself, and ANS activity can also differ depending on sex, the time of measurement, and whether the animals are under general anaesthesia. Thus, for correct assessment, changes in ANS activity and their relationship to the observed parameter should be based on whether ANS activity does or does not change but also to what extent the activity is already changed at the start of the experiment. Since rats are considered to be the most suitable model animal for basic cardiovascular research, in this review we point out existing differences in individual HRV frequency parameters at the start of experiments (control, baseline values), taking into account sex in relation to time of measurement and anaesthesia.

## Abstract (highlights)

- **What is the topic of this review?**
  Changes in heart rate variability in rats with sex differences and the use of different anaesthesia during light–dark cycles.
- **What advances does it highlight?**
  The review highlights and discusses synthesized current results in order to advance knowledge and understanding of sex differences with an emphasis on changes in the autonomic nervous system determined by heart rate variability.

## 1 INTRODUCTION

The autonomic nervous system (ANS) has a dominant role in the regulation of vital functions in the organism. As such, the correct evaluation of changes in sympathetic and parasympathetic activity linked to an observed parameter is of importance for the assessment of their mutual interactions. Because the ANS in rats is very sensitive to external interventions, such as animal handling, experimental methodology, surgical intervention(s), recovery time after surgical intervention(s), and/or the use of general anaesthesia, the activity of the ANS can be significantly altered even before the beginning of an experiment, which can result in misinterpretation of the final results.

Another factor that can affect the ANS is sex, which is supported by studies showing that there are differences in the regulatory mechanisms of the cardiovascular system that depend on sex. An example could be the finding that males exhibited worse survival in a heart failure model with preserved ejection fraction and had a higher risk of sudden death than females (Elkholey et al., 2021). Experimental data from animal models strongly support the concept that sex‐dependent variation must also be taken into account, in which female humans and female animals use different mechanisms to regulate the cardiovascular system (Meyer et al., 2006). If there are different mechanisms of regulation of the cardiovascular system and if the cardiovascular system is predominantly regulated by the ANS, it logically follows that there must also be sex differences in the activities of the individual divisions of the ANS.

If ANS activity is assessed on the basis of changes in cardiovascular parameters, then all measurable cardiovascular parameters that exhibit fluctuations during a 24 h period (i.e., circadian rhythm) must also be taken into account. For this reason, synchronization of the animals to the light–dark (LD) cycle, in which the experiments are performed in the light period, or specifying the time of day when the measurements are made, is important. Significant diurnal variations in heart rate (HR) and blood pressure have been found in normotensive as well as spontaneously hypertensive female and male rats. HR was higher in normotensive females compared to all other groups, whereas spontaneously hypertensive males exhibited higher systolic blood pressure than females (Maris et al., 2005). Under conditions of an alternating 12:12 h LD cycle, HR was higher in pro‐oestrus females than in spontaneously hypertensive males, while blood pressure was inversely higher in males than in females (Grund et al., 2006). The question of whether cardiorespiratory functions are controlled by the endogenous circadian system and whether they change with the oestrous cycle in female rats was the aim of a study by Takezawa et al. (1994), who measured circadian rhythms in mean arterial pressure, HR and spontaneous activity using an implanted radiotelemetry device. Based on the results, the authors concluded that the daily variations in the mentioned parameters are controlled by the endogenous circadian oscillatory system, which changes with the oestrous cycle in female rats, and that oestrogen may be responsible for these variations dependent on the oestrous cycle.

For these reasons, all factors that can affect the state of the ANS before experimentation and to what extent the control (baseline) values themselves can be changed by these factors should be carefully considered when designing experiments.

## 2 CHRONOBIOLOGY OF THE ANS IN RATS

In addition to humans, all animals are exposed to periodic cycles of alternating light and dark during the 24 h (i.e., circadian) period, with which all physiological functions are synchronized. Disruption of the internal synchronization of rhythms with the periodicity of the external environment (circadian disruption) can manifest itself in an increased susceptibility to disease(s). Therefore, creating experimental, in vivo chronobiological animal models can help reveal some relationships between circadian time and biological function, which are sometimes very difficult to study in humans.

Many studies and various experimental animal models have addressed the phenomenon of circadian fluctuations in the activities of the ANS system. Studies involving rats clearly point to this rhythmicity by monitoring the circadian rhythms of some cardiovascular parameters. Even in the previous century, some authors carefully reported that the ANS does not have direct control over the circadian rhythmicity of blood pressure and HR in the rat, but only has a modulating influence (Makino et al., 1997). Makino et al. (1997) demonstrated that disruption of the baroreceptor reflex system in rats selectively eliminated the circadian rhythm of blood pressure due to an increase in blood pressure during the light (i.e., inactive) period, and that the circadian rhythms of blood pressure and HR are regulated by different mechanisms, including the ANS. The results also indicate that in rats sympathetic nervous system blockade suppresses circadian rhythms of blood pressure and HR by decreasing blood pressure and HR during the dark (i.e., active) period, and blockade of the parasympathetic system suppresses circadian rhythms of blood pressure and HR by increasing blood pressure and HR during the light period of the LD cycle (Makino et al., 1997).

The activity of the ANS fluctuates depending on the LD cycle of the rat daily regimen. Rapid control of HR is accomplished primarily through a balance between vagal and sympathetic activity innervating the sinoatrial node (Dampney, 1994; Spyer, 1992). Light onset‐induced changes in heart rate variability (HRV) parameters in rats are interpreted as a greater increase in sympathetic activity than in vagal activity due to a shift in the low frequency (LF)/high frequency (HF) ratio. LF components have been attributed to vasomotor‐driven thermoregulation and the renin–angiotensin system (Malliani et al., 1991; Spyer, 1992).

Further evidence supporting the direct control of the cardiovascular system by the ANS was published by Hashimoto et al. (1999), who described the results of experiments that monitored circadian oscillations in HR and HRV. HR values were higher in the dark (20.00−06.00 h) compared with those in the light (06.00−20.00 h) period. LF, in addition to HF, components of HRV were higher in the light than in the dark period; however, these differences were not statistically significant. The LF/HF ratio reflects LD differences in male rats, with significantly lower values during the light (inactive) period of the day. LF and HF power appear to be predominantly affected in the light period compared to the dark period. These results suggest that sympathetic nerve activity predominates in the dark phase in rats (Hashimoto et al., 2001, 1999, 2001, 1999). Although the synchronization of animals to the LD cycle was not specified in the study by Mamalyga (2014), the authors reported that total spectral power was significantly increased in the morning hours (08.00−12.00 h), while sympathetic activity prevailed in each evaluated interval. Further confirmation of the existence of 24 h fluctuations in the activities of the ANS in the rat is reported in the results of other studies (Farraj et al., 2009; Johnson et al., 2011; Koresh et al., 2016). Although the results are not unambiguous, comparisons of individual spectral powers and LF/HF ratio indicate differences between the light and dark periods of the rat daily regimen.

Similar results were reported by Towa et al. (2004), who recorded diurnal variation of locomotor activity and HRV in lean and obese rats after synchronizing the animals to a 12:12 h LD cycle, with the light period from 08.00 to 20.00 h. There were no significant differences in HF, LF and LF/HF parameters between obese and lean rats, while the circadian rhythm of these parameters was mostly preserved in both strains of rats. LF and HF components during the light period were significantly higher than during the dark period. In awake, freely moving rats, Ohori et al. (2011) demonstrated that the LF component of diastolic blood pressure, an index of sympathetic tone, was significantly higher during the wakeful period (16.00−20.00 h) than during sleep (08.00−14.00 h). Baseline values of HR, mean blood pressure and locomotor activity were higher in the dark period than in the light period, even in the sham group of animals (i.e., rats that underwent thoracotomy but not coronary artery ligation), which is consistent with the circadian periodicity of nocturnal animals.

HR, LF, HF, and LF/HF ratio measured 3 days after surgery required for implantation of a telemetry transmitter exhibited a clear circadian rhythm, with higher values during the dark period and lower values during the light period of the rats’ routine day. The LF/HF ratio also exhibited a circadian rhythm. The LF/HF ratio was high during the dark part of the day and low during the light part of the day (Ohnuki et al., 2001). Albarwani et al. (2013) monitored HRV 5 days after surgery for 10 weeks in spontaneously hypertensive male rats at 07.00 h during the day (inactive period) and at 19.00 h (active period) at night. In the control group, HF components were significantly higher in the inactive than in the active period; LF and very low frequency (VLF) components were significantly lower and did not differ significantly between the two light periods. The LF/HF ratio reflected the dominance of the parasympathetic system, especially in the inactive part of the day. Similar results were obtained with spontaneously hypertensive rats in a study by Wessel et al. (2007). These results indicate the predominance of different mechanisms of neuroautonomical regulation of heart rhythm at different hours of the day, which largely determines the circadian dynamics of the functional manifestations of this condition.

Direct circadian control of blood pressure and HR in rats by the suprachiasmatic nuclei in the hypothalamus was also confirmed. It is now known that the suprachiasmatic nuclei are mammalian biological clocks that generate daily rhythms in physiology and behaviour. This fact was confirmed by experiments in which the effect of a possible functional neural connection of the circadian pacemaker in the hypothalamus on the peripheral organs was monitored. Although light can cause a phase shift in the rhythm of the suprachiasmatic nuclei, it can also acutely affect the activity of the suprachiasmatic nuclei and the output itself (e.g., output to the pineal gland). HR in intact male Wistar rats – but not rats with lesions of the suprachiasmatic nuclei – was found to exhibit a clear circadian rhythm that was not due to locomotor activity. Light at night has been shown to decrease HR in intact rats but not in rats with lesions of the suprachiasmatic nuclei (Scheer et al., 2001). Circadian disruption significantly increased blood pressure, renal sympathetic nerve activity and plasma noradrenaline levels (Duan et al., 2022). The above examples clearly provide evidence supporting the existence of circadian rhythms in ANS activity in male rats. The question is whether similar circadian variations exist in female rats.

## 3 HR VARIABILITY IN CONSCIOUS FEMALE RATS

Sex is usually not considered in cardiovascular and toxicological in vivo experiments involving rats, although this type of experimental animal model is commonly used to investigate normal and pathological physiology. Most experimental studies use only male rats; however, in females there are differences from males in essential functional systems, and responses to the same interventions differ from those in males.

In an attempt to assess to what extent sex differences in rat HRV were addressed in the international scientific literature, of 496 articles retrieved from the Web of Science database using the keywords 'HRV in rats', only four described HRV, and two chronobiological studies involving conscious female rats.

Although hypotheses regarding sex differences in the activities of the ANS measured according HRV have not yet been confirmed, they have not been rejected because there are only four studies that involved conscious female rats (Johnson et al., 2011; Koresh et al., 2016; Ramadoss et al., 2016; Singh et al., 2018) whereas the control values from individual studies are not unequivocal. For example, in a study by Johnson et al. (2011), LF was higher than HF spectral power, which would indicate the predominance of baroreceptor activity. In a study by Ramadoss et al. (2016), the values of frequency parameters were significantly higher compared to other studies and, therefore, were not included in the average values presented in Table 1, and the LF/HF ratio indicates the balance between the sympathetic and parasympathetic systems. VLF spectral power has not been evaluated. Overall, averaged control values of the HF spectral power as well as the LF/HF ratio indicate a dominance of parasympathetic activity. When comparing LF/HF ratio in the light (i.e., inactive) period, there are probably sex differences, in which the sympathetic system predominates in males and the parasympathetic system in females.

**TABLE 1:** Averaged numerical values of heart rate variability (HRV) frequency parameters in conscious females as well as values from individual studies from the light period of the rat daily regimen.

Reference	VLF power	LF power	HF power	LF/HF
Male	19.35 (*n* = 5)	4.72 (*n* = 10)	5.19 (*n* = 11)	1.66 (*n* = 19)
Female	3.8 (*n* = 1)	2.73 (*n* = 3)	3.52 (*n* = 3)	0.37 (*n* = 2)
Singh et al. (2018)		1.81 ± 4.2	4.75 ± 12.85	0.44 ± 0.37
Johnson et al. (2011)	3.8 ± 1.30	4.1 ± 1.09	2.5 ± 0.63	
Koresh et al. (2016)		2.29 ± 0.06	3.3 ± 0.03	0.3 ± 0.006
Ramadoss et al. (2016)		15.07 ± 1.9	15.87 ± 4.03	0.98 ± 0.17

VLF power: VLF spectral power of HRV; LF power: LF spectral power of HRV; HF power: HF spectral power of HRV – presented in ms^2^. Male: average values of individual frequency parameters calculated from studies with telemetric recording in awake male rats; *n*: number of studies from which HRV parameters were evaluated. Female: average values of individual frequency parameters calculated from studies with telemetric recording in awake female rats; n: number of studies from which HRV parameters were evaluated.

## 4 SEX AND CHRONOBIOLOGY OF HRV IN CONSCIOUS RATS

After 'averaging' the results of HRV parameters from conscious rats of both sexes, which also accounted for chronobiological principles (Johnson et al., 2011; Koresh et al., 2016), we speculatively conclude that there are probably sex‐based HRV differences, although the differences are not statistically significant. The sympathovagal balance (LF/HF) is shifted to parasympathetic dominance with a lower value in females in both light and dark periods. The values of LF and HF components of HRV are ambiguous, where LF power (sympathetic and parasympathetic activity, baroreflex, baroreceptor activity) in females is higher in the light period, but lower in the dark period compared to males, and HF power (parasympathetic activity) is higher in both light and dark periods in males versus females.

The baseline, or control, averaged values of some parameters of the frequency‐domain analysis, obtained from awake female and male rats by telemetry, taking into account the LD cycle, are summarized in Table 2. Unfortunately, there are no time‐domain analysis parameters available. Because only a few experiments have been conducted in this way, the values are only indicative and the values given are not relevant, because there were only two comparative studies (Johnson et al., 2011; Koresh et al., 2016).

**TABLE 2:** Sex differences in cardiac autonomic nerve control.

Variable, reference(s)	Female		Male	
	Light	Dark	Light	Dark
VLF, Johnson et al. (2011)	3.8	4.5	5.0	5.4
	2.5–5.1	4.02–4.98	3.15–6.85	4.24–6.56
LF, Johnson et al. (2011), Koresh et al. (2016)	3.2	2.99	2.88	3.01
	2.26–3.77	2.66–3.32	2.15–3.25	2.65–3.37
HF, Johnson et al. (2011), Koresh et al. (2016)	2.65	2.4	3.43	2.82
	2.57–3.23	2.24–2.55	2.88–3.99	2.39–3.25
LF/HF, Koresh et al. (2016)	0.3	0.39	0.41	0.52
	0.294–0.306	0.383–0.397	0.404–0.416	0.514–0.526

Averaged values and ranges of some heart rate variability frequency parameters from two studies in conscious rats of both sexes, in which chronobiological principles were accepted. HF, high frequency; LF, low frequency; VLF, very low frequency.

## 5 SEX AND CHRONOBIOLOGY OF HRV UNDER GENERAL ANAESTHESIA

It is generally accepted that general anaesthesia reduces HRV, which cannot be relevantly assessed from our analysis because there are few studies involving female rats; however, it is clear from the results that parasympathetic activity dominated in females under ketamine/xylazine (Gonçalves et al., 2010; Elmas & Comlekci, 2015; Svorc et al., 2014) as well as under pentobarbital anaesthesia (Svorc et al., 2015).

In experiments in which chronobiological principles were accepted, in ketamine/xylazine‐anesthetized female rats, significant LD differences were found in all HRV spectral powers, with higher values during the light period (Table 3). The spectral powers of individual HRV parameters correspond to the dominant influence of the parasympathetic (power HF) division of the ANS, with a moderate influence of baroreceptor (power LF) and sympathetic activity (power VLF) in both light periods of the daily regimen, but without significant LD differences (Svorc et al., 2014). On the other hand, pentobarbital anaesthesia eliminated LD differences in all HRV spectral powers (except RR intervals), whereas, similar to ketanine/xylazine anaesthesia, all HRV parameters were higher in the light period (Svorc et al., 2015).

**TABLE 3:** Mean numerical values of heart rate variability (HRV) frequency parameters under general anaesthesia in females.

Author; anaesthesia	VLF power	LF power	HF power	LF/HF	LF n.u.	HF n.u.
Gonçalves et al. (2010); ketamine		11.05 ± 1.95	24.85 ± 5.35	0.54 ± 0.095		
Elmas & Comlekci, 2015; K/X				0.39 ± 0.27	21.6 ± 6.0	62.5 ± 16.8
Svorc et al. (2014); K/X						
Light	0.091 ± 0.04	0.182 ± 0.01	5.537 ± 2.34			
Dark	0.045 ± 0.02	0.107 ± 0.06	1.324 ± 0.59			
Svorc et al. (2015); pentobarbital						
Light		0.139 ± 0.19	1.415 ± 1.36	0.137 ± 0.15		
Dark		0.111 ± 0.17	1.097 ± 0.8	0.126 ± 0.15		

VLF power: VLF spectral power of HRV; LF power: LF spectral power of HRV; HF power: HF spectral power of HRV – in ms^2^; LF n.u. and HF n.u.: HRV frequency parameters expressed in normalized units. HF, high frequency; LF, low frequency; VLF, very low frequency; K/X, ketamine/xylazine.

**FIGURE 1:**
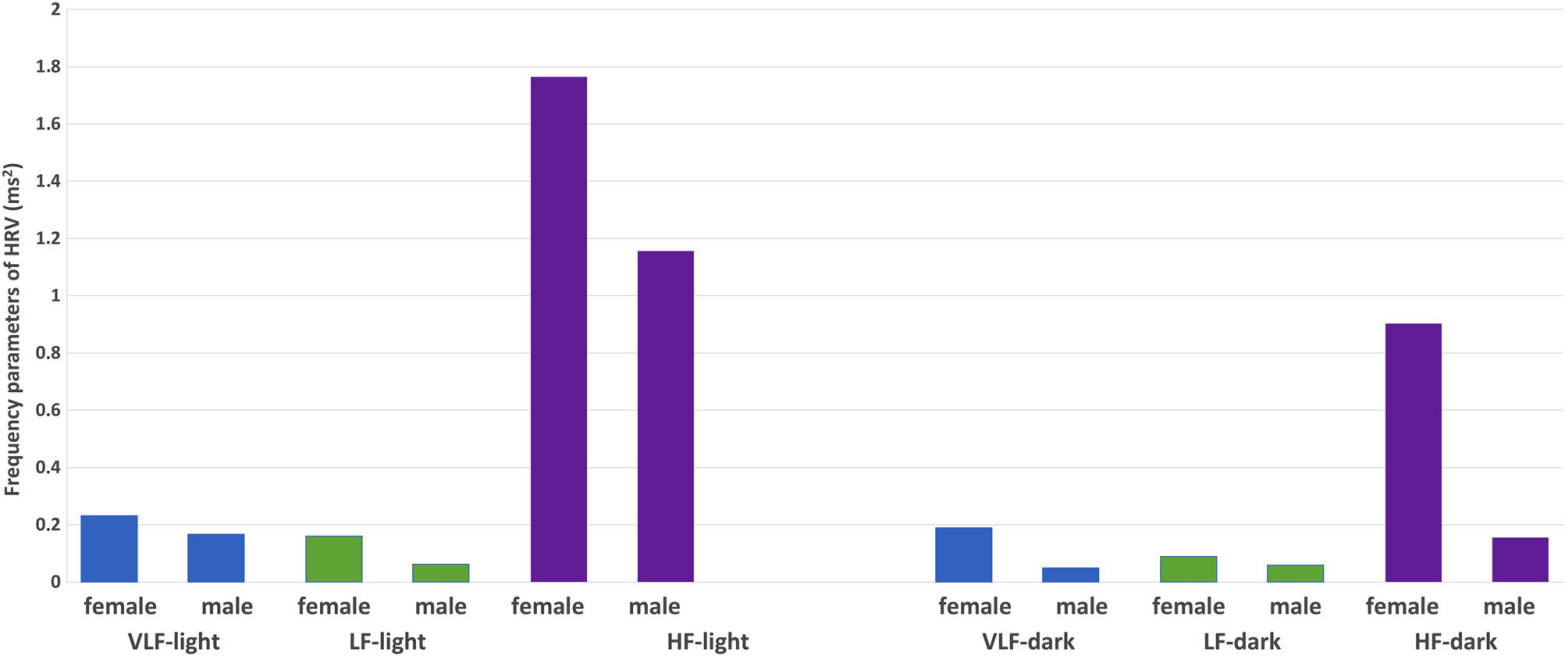
Graphical representation of sex differences in individual heart rate variability (HRV) parameters depending on the light–dark (LD) cycle in rats under zoletil anaesthesia.

Another possible indication of the existence of sex differences in the activities of the ANS is evident from experiments involving zoletil anaesthesia. The results show that with this type of anaesthesia, although sympathetic (VLF) and baroreceptor (LF) activity is reduced compared to parasympathetic (HF) activity in both sexes and in both light periods, there are sex differences depending on the LD cycle. Taking sex differences into account, the LF/HF ratio reflects more pronounced parasympathetic activity in males than in females in the light part of the rat daily regimen. In the dark part of the day, parasympathetic activity is increased in females compared to males (Table 4). During both the light and dark period of the rat daily regimen, all observed HRV frequency parameters were higher in females compared to males. LD differences were preserved mainly in HF power; thus, the circadian rhythm in parasympathetic activity probably persists in both sexes. It was concluded that sex differences exist in rat ANS activity, persist under general anaesthesia, and depend on the LD cycle, which may also be related to its regulatory effect on the cardiovascular system (Figure 1) (Svorc et al., 2020). Although this was a study investigating zoletil anaesthesia, it is likely that such sex differences can also occur in awake rats. During pentobarbital, ketamine/xylazine and zoletil anaesthesia, parasympathetic tone prevails in both light periods with reduced sympathetic activity.

**TABLE 4:** Sex differences in the frequency parameters of heart rate variability (HRV) in zoletil anesthetized rats depending on light‐dark (LD) cycle.

Variable	Female		Male	
	Light	Dark	Light	Dark
VLF	0.232 ± 0.26	0.189 ± 0.24	0.167 ± 0.19	0.049 ± 0.02
LF	0.16 ± 0.22	0.089 ± 0.16	0.062 ± 0.05	0.059 ± 0.11
HF	1.765 ± 2.28	0.903 ± 0.73	1.156 ± 0.87	0.155 ± 0.09
LF/HF	0.34 ± 0.39	0.113 ± 0.17	0.105 ± 0.13	0.28 ± 0.198

Data presented as means ± standard deviation. HF, high frequency; LF, low frequency; VLF, very low frequency.

## 6 HRV AND HR

The relationship between HRV and HR in terms of sex in rats is also interesting. HR is an easily measured parameter of cardiac activity, and changes in HR can have a direct impact on the cardiovascular system. Caetano and Alves (2015) reported that elevated resting HR is an independent predictor of cardiovascular and total mortality, and the occurrence of arrhythmias is, thus, often associated with baseline HR, which has prognostic significance (Zaza et al., 2018). It can, therefore, be assumed that the initial HR in in vivo cardiovascular studies can significantly influence the results obtained during the experiment, what is also valid for rats (Baldwin et al., 2008; Heisser, 2020; Hannon, 1958).

HR and HRV are not independent variables because HR is influenced by sympathovagal balance (Bootsma et al., 1994; Coumel et al., 1994; Malik & Camm, 1993) and thus is the result of mutual interactions between parasympathetic and sympathetic activity. However, HR exhibits circadian fluctuations, and values measured at specific times of the day should reasonably be accepted. The reason for such an approach is the fact that telemetry studies in awake rats indicate the existence of a circadian rhythm in HR, not only in males (Hashimoto et al., 2001, 1999, 2001, 1999; Koresh et al., 2016; Molcan et al., 2013, 2014, 2013, 2014; Wessel et al., 2007) but also in females (Koresh et al., 2016; Schlatter & Zbinden, 1982). Therefore, in this regard, the use of the HRV method can be an effective and non‐invasive tool for the assessment of autonomic control of the heart, as well as autonomic modulation of HR (Akselrod, 1988; Lunqvist, 1990; Malik & Camm, 1993; Mansier et al., 1996), for which changes are a useful indicator of tendencies toward heart rhythm disorders. A positive relationship between the RR interval and various HRV parameters has been demonstrated: the slower the HR, the greater the HRV. Nevertheless, studies investigating the relationships between HR and HRV remain rare, and devoting more attention to the complex relationships between HR and HRV, even in gender differences.

Sex‐based differences have been found in studies analysing the dependency of HR on sympathetic and parasympathetic activities. Experiments were performed using zoletil anaesthesia, in which it was found that in males, significant LD fluctuations in HR were preserved and changes in HR exhibited no dependence on ANS activity in both light periods; therefore, it is assumed that they are determined by other factors. In females, changes in HR were significantly dependent on ANS activity in both light periods (Svorc et al., 2020, 2022).

## 7 CONCLUSIONS

Based on the results presented herein, we believe drawing definitive conclusions is a problem. Although there are very few studies investigating sex differences in the activities of the ANS in rats, sex differences probably exist. It seems that in awake females, parasympathetic activity dominates more significantly than in males, in both light periods of the regime day.

When designing an experiment, the time when the measurements are performed (the existence of circadian rhythms) and, if the experiments are performed under general anaesthesia, the type of aesthetic used should be taken into account. General anaesthesia reduces HRV in females in both light periods. However, the presented results are from the light (inactive) period, so it is necessary to make measurements also from the dark (active) period of the rat daily regimen.

Unfortunately, while this cannot be assessed from our analysis because there are few studies involving female rats, the results indicate the predominance of different mechanisms of neuroautonomical regulation of heart rhythm in males and females and many more studies are required in the future to confirm these differences.

## AUTHOR CONTRIBUTIONS

Pavol Svorc proposed the original draft of the study. Pavol Svorc Jr, Sona Gresova collected all data and performed data analysis. Pavol Svorc designed the first manuscript. Pavol Svorc and Sona Gresova drafted the final manuscript. All authors made critical revisions and approved the final version of the manuscript. All authors agree to be accountable for all aspects of the work in ensuring that questions related to the accuracy or integrity of any part of the work are appropriately investigated and resolved. All persons designated as authors qualify for authorship, and all those who qualify for authorship are listed.

## CONFLICT OF INTEREST

None declared.

## FUNDING INFORMATION

None.

## References

[eph13342-bib-0001] Akselrod, S. (1988). Spectral analysis of fluctuations in cardiovascular parameters: A quantitative tool for the investigation of autonomic control. Trends in Pharmacological Sciences, 9(1), 6–9. 3245076 10.1016/0165-6147(88)90230-1

[eph13342-bib-0002] Albarwani, S. , Al‐Siyabi, S. , & Tanira, M. O. (2013). Lisinopril indifferently improves heart rate variability during day and night periods in spontaneously hypertensive rats. Physiological Research, 62(3), 237–245. 23489185 10.33549/physiolres.932425

[eph13342-bib-0003] Baldwin, A. L. , Wagers, C. , & Schwartz, G. E. (2008). Reiki improves heart rate homeostasis in laboratory rats. Journal of Alternative and Complementary Medicine, 14(4), 417–422. 18435597 10.1089/acm.2007.0753

[eph13342-bib-0004] Bootsma, M. , Swenne, C. A. , Van Bolhuis, H. H. , Chang, P. C. , Cats, V. M. , & Bruschke, A. V. (1994). Heart rate and heart rate variability as indexes of sympathovagal balance. American Journal of Physiology, 266(4 Pt 2), H1565–H1571. 8184935 10.1152/ajpheart.1994.266.4.H1565

[eph13342-bib-0005] Caetano, J. , & Delgado Alves, J. (2015). Heart rate and cardiovascular protection. European Journal of Internal Medicine, 26(4), 217–222. 25704330 10.1016/j.ejim.2015.02.009

[eph13342-bib-0006] Coumel, P. , Maison‐Blanche, P. , & Catuli, D. (1994). Heart rate and heart rate variability in normal young adults. Journal of Cardiovascular Electrophysiology, 5(11), 899–911. 7889230 10.1111/j.1540-8167.1994.tb01130.x

[eph13342-bib-0007] Dampney, R. A. (1994). Functional organization of central pathways regulating the cardiovascular system. Physiological Reviews, 74(2), 323–364. 8171117 10.1152/physrev.1994.74.2.323

[eph13342-bib-0008] Duan, W. , Ye, P. , Leng, Y. Q. , Liu, D. H. , Sun, J. C. , Tan, X. , & Wang, W. Z. (2022). Oxidative stress in the RVLM mediates sympathetic hyperactivity induced by circadian disruption. Neuroscience Letters, 791, 136917. 36252850 10.1016/j.neulet.2022.136917

[eph13342-bib-0009] Elkholey, K. , Morris, L. , Niewiadomska, M. , Houser, J. , Ramirez, M. , Tang, M. , Humphrey, M. B. , & Stavrakis, S. (2021). Sex differences in the incidence and mode of death in rats with heart failure with preserved ejection fraction. Experimental Physiology, 106(3), 673–682. 33428276 10.1113/EP089163 PMC7920931

[eph13342-bib-0010] Elmas, O. , & Comlekci, S. (2015). Investigation of effects of short‐term exposure to 50 HZ magnetic field on central, peripheral, and autonomic nervous systems in rats. Bioelectromagnetics, 36(6), 420–429. 25974832 10.1002/bem.21922

[eph13342-bib-0011] Farraj, A. K. , Haykal‐Coates, N. , Winsett, D. W. , Hazari, M. S. , Carll, A. P. , Rowan, W. H. , Ledbetter, A. D. , Cascio, W. E. , & Costa, D. L. (2009). Increased non‐conducted P‐wave arrhythmias after a single oil fly ash inhalation exposure in hypertensive rats. Environmental Health Perspectives, 117(5), 709–715. 19479011 10.1289/ehp.0800129 PMC2685831

[eph13342-bib-0012] Gonçalves, H. , Henriques‐Coelho, T. , Bernardes, J. , Rocha, A. P. , Brandão‐Nogueira, A. , & Leite‐Moreira, A. (2010). Analysis of heart rate variability in a rat model of induced pulmonary hypertension. Medical Engineering & Physics, 32(7), 746–752. 20547091 10.1016/j.medengphy.2010.04.018

[eph13342-bib-0013] Grundt, C. , Meier, K. , & Lemmer, B. (2006). Gender dependency of circadian blood pressure and heart rate profiles in spontaneously hypertensive rats: Effects of beta‐blockers. Chronobiology International, 23(4), 813–829. 16887750 10.1080/07420520600827129

[eph13342-bib-0014] Hannon, J. P. (1958). Effect of temperature on the heart rate, electrocardiogram and certain myocardial oxidations of the rat. Circulation Research, 6(6), 771–778. 13585606 10.1161/01.res.6.6.771

[eph13342-bib-0015] Hashimoto, M. , Harada, T. , Ishikawa, T. , Obata, M. , & Shibutani, Y. (2001). Investigation on diabetic autonomic neuropathy assessed by power spectral analysis of heart rate variability in WBN/Kob rats. Journal of Electrocardiology, 34(3), 243–250. 10.1054/jelc.2001.25130 11455515

[eph13342-bib-0016] Hashimoto, M. , Kuwahara, M. , Tsubone, H. , & Sugano, S. (1999). Diurnal variation of autonomic nervous activity in the rat: Investigation by power spectral analysis of heart rate variability. Journal of Electrocardiology, 32(2), 167–171. 10338035

[eph13342-bib-0017] Heisser, A. (2020). Effect of exercise and L‐citrulline on heart rate in rats. Cantaurus, 28, 5–7.

[eph13342-bib-0018] Johnson, M. S. , DeMarco, V. G. , Heesch, C. M. , Whaley‐Connell, A. T. , Schneider, R. I. , Rehmer, N. T. , Tilmon, R. D. , Ferrario, C. M. , & Sowers, J. R. (2011). Sex differences in baroreflex sensitivity, heart rate variability, and end organ damage in the TGR(mRen2)27 rat. American Journal of Physiology. Heart and Circulatory Physiology, 301(4), H1540–H1550. 21821781 10.1152/ajpheart.00593.2011 PMC3197369

[eph13342-bib-0019] Koresh, O. , Kaplan, Z. , Zohar, J. , Matar, M. A. , Geva, A. B. , & Cohen, H. (2016). Distinctive cardiac autonomic dysfunction following stress exposure in both sexes in an animal model of PTSD. Behavioural Brain Research, 308, 128–142. 27105958 10.1016/j.bbr.2016.04.024

[eph13342-bib-0020] Lindqvist, A. (1990). Noninvasive methods to study autonomic nervous control of circulation. Acta physiologica Scandinavica. Supplementum, 588, 1–107. 2192535

[eph13342-bib-0021] Makino, M. , Hayashi, H. , Takezawa, H. , Hirai, M. , Saito, H. , & Ebihara, S. (1997). Circadian rhythms of cardiovascular functions are modulated by the baroreflex and the autonomic nervous system in the rat. Circulation, 96(5), 1667–1674. 9315563 10.1161/01.cir.96.5.1667

[eph13342-bib-0022] Malik, M. , & Camm, A. J. (1993). Components of heart rate variability–what they really mean and what we really measure. The American Journal of Cardiology, 72(11), 821–822. 8093124 10.1016/0002-9149(93)91070-x

[eph13342-bib-0023] Malliani, A. , Pagani, M. , Lombardi, F. , & Cerutti, S. (1991). Cardiovascular neural regulation explored in the frequency domain. Circulation, 84(2), 482–492. 1860193 10.1161/01.cir.84.2.482

[eph13342-bib-0024] Mamalyga, M. L. (2014). Circadian changes in cardiac rhythm structure in decompensated chronic heart failure. Bulletin of Experimental Biology and Medicine, 156(4), 499–503. 24771437 10.1007/s10517-014-2384-5

[eph13342-bib-0025] Mansier, P. , Clairambault, J. , Charlotte, N. , Médigue, C. , Vermeiren, C. , LePape, G. , Carré, F. , Gounaropoulou, A. , & Swynghedauw, B. (1996). Linear and non‐linear analyses of heart rate variability: A minireview. Cardiovascular Research, 31(3), 371–379. 8681324

[eph13342-bib-0026] Maris, M. E. , Melchert, R. B. , Joseph, J. , & Kennedy, R. H. (2005). Gender differences in blood pressure and heart rate in spontaneously hypertensive and Wistar‐Kyoto rats. Clinical and Experimental Pharmacology & Physiology, 32(1‐2), 35–39. 15730432 10.1111/j.1440-1681.2005.04156.x

[eph13342-bib-0027] Meyer, M. R. , Haas, E. , & Barton, M. (2006). Gender differences of cardiovascular disease: New perspectives for estrogen receptor signaling. Hypertension, 47(6), 1019–1026. 16651458 10.1161/01.HYP.0000223064.62762.0b

[eph13342-bib-0028] Molcan, L. , Teplan, M. , Vesela, A. , & Zeman, M. (2013). The long‐term effects of phase advance shifts of photoperiod on cardiovascular parameters as measured by radiotelemetry in rats. Physiological Measurement, 34(12), 1623–1632. 24165479 10.1088/0967-3334/34/12/1623

[eph13342-bib-0029] Molcan, L. , Vesela, A. , & Zeman, M. (2014). Repeated phase shifts in the lighting regimen change the blood pressure response to norepinephrine stimulation in rats. Physiological Research, 63(5), 567–575. 24908081 10.33549/physiolres.932653

[eph13342-bib-0045] Ohnuki, K. , Moritani, T. , Ishihara, K. , & Fushiki, T. (2001). Capsaicin increases modulation of sympathetic nerve activity in rats: measurement using power spectral analysis of heart rate fluctuations. Bioscience, biotechnology, and biochemistry, 65(3), 638–643. 11330680 10.1271/bbb.65.638

[eph13342-bib-0030] Ohori, T. , Hirai, T. , Joho, S. , Kameyama, T. , Nozawa, T. , Asanoi, H. , & Inoue, H. (2011). Circadian changes in autonomic function in conscious rats with heart failure: Effects of amiodarone on sympathetic surge. Autonomic Neuroscience: Basic & Clinical, 159(1‐2), 20–25. 20674512 10.1016/j.autneu.2010.07.001

[eph13342-bib-0031] Ramadoss, M. , Ramanathan, G. , Subbiah, A. J. , & Natrajan, C. (2016). Heart rate changes in electroacupuncture treated polycystic ovary in rats. Journal of Clinical and Diagnostic Research, 10(3), CF01–CF3. 10.7860/JCDR/2016/18303.7395 PMC4843254 27134868

[eph13342-bib-0032] Scheer, F. A. , Horst, T. , G, J. , van Der Vliet, J. , & Buijs, R. M. (2001). Physiological and anatomic evidence for regulation of the heart by suprachiasmatic nucleus in rats. American Journal of Physiology. Heart and Circulatory Physiology, 280(3), H1391–H1399. 11179089 10.1152/ajpheart.2001.280.3.H1391

[eph13342-bib-0033] Schlatter, J. , & Zbinden, G. (1982). Heart rate‐ and ECG‐recording in the rat by biotelemetry. Archives of Toxicology. Supplement = Archiv fur Toxikologie. Supplement, 5, 179–183. 6954896 10.1007/978-3-642-68511-8_31

[eph13342-bib-0034] Singh, N. M. , Sathyaprabha, T. N. , Malthish, K. , Thirthalli, J. , & Andrade, C. (2018). Early and late postictal cardiac electrophysiological changes associated with low, moderate, and high dose electroconvulsive shocks. Asian Journal of Psychiatry, 33, 78–83. 29547752 10.1016/j.ajp.2018.03.001

[eph13342-bib-0035] Spyer, K. M. (1992). Central nervous control of the cardiovascular system. Autonomic Failure. A Text‐book of Clinical Disorders of the Autonomic Nervous System, 54–77.

[eph13342-bib-0036] Svorc, P. , Bacova, I. , & Gresova, S. (2015). Pentobarbital anaesthesia in the chronobiological studies. Biological Rhythm Research, 46(3), 445–452.

[eph13342-bib-0037] Svorc, P. , Novakova, M. , Bacova, I. , Jurasova, Z. , & Marossy, A. (2014). Ketamine/xylazine anaesthesia in the chronobiological studies. Biological Rhythm Research, 45(4), 633–642.

[eph13342-bib-0038] Svorc, P. , Petrasova, D. , & Svorc Jr, P. (2020). Sex differences in HRV under general anesthesia in rat model. Anesthesia and Pain Medicine, 4(1), 1–6.

[eph13342-bib-0039] Svorc, P. , Petrasova, D. , & Svorc Jr, P. (2022). Study on heart rate variability and heart rate under general anesthesia in rats of both sexes. Chapter 2. In: Issues and Developments in Medicine and Medical Research, 5, 17–22. Print ISBN: 978‐93‐5547‐463‐6, eBook ISBN: 978‐93‐5547‐479‐7. 10.9734/bpi/idmmr/v5/2413C

[eph13342-bib-0040] Takezawa, H. , Hayashi, H. , Sano, H. , Saito, H. , & Ebihara, S. (1994). Circadian and estrous cycle‐dependent variations in blood pressure and heart rate in female rats. American Journal of Physiology, 267(5 Pt 2), R1250–R1256. 7977852 10.1152/ajpregu.1994.267.5.R1250

[eph13342-bib-0041] Towa, S. , Kuwahara, M. , & Tsubone, H. (2004). Characteristics of autonomic nervous function in Zucker‐fatty rats: Investigation by power spectral analysis of heart rate variability. Experimental Animals, 53(2), 137–144. 15153676 10.1538/expanim.53.137

[eph13342-bib-0042] Wessel, N. , Malberg, H. , Heringer‐Walther, S. , Schultheiss, H. P. , & Walther, T. (2007). The angiotensin‐(1‐7) receptor agonist AVE0991 dominates the circadian rhythm and baroreflex in spontaneously hypertensive rats. Journal of Cardiovascular Pharmacology, 49(2), 67–73. 17312445 10.1097/FJC.0b013e31802cffe9

[eph13342-bib-0043] Zaza, A. , Ronchi, C. , & Malfatto, G. (2018). Arrhythmias and heart rate: Mechanisms and significance of a relationship. Arrhythmia & Electrophysiology Review, 7(4), 232–237. 30588310 10.15420/aer.2018.12.3 PMC6304796

